# Relationship between Vehicle Emissions Laws and Incidence of Suicide by Motor Vehicle Exhaust Gas in Australia, 2001–06: An Ecological Analysis

**DOI:** 10.1371/journal.pmed.1000210

**Published:** 2010-01-05

**Authors:** David M. Studdert, Lyle C. Gurrin, Uma Jatkar, Jane Pirkis

**Affiliations:** 1 Melbourne School of Population Health, University of Melbourne, Melbourne, Australia; 2 Melbourne Law School, University of Melbourne, Melbourne, Australia; King‚s College London, United Kingdom

## Abstract

**Background:**

Globally, suicide accounts for 5.2% of deaths among persons aged 15 to 44 years and its incidence is rising. In Australia, suicide rates peaked in 1997 and have been declining since. A substantial part of that decline stems from a plunge in suicides by one particular method: asphyxiation by motor vehicle exhaust gas (MVEG). Although MVEG remains the second most common method of suicide in Australia, its incidence decreased by nearly 70% in the decade to 2006. The extent to which this phenomenon has been driven by national laws in 1986 and 1999 that lowered permissible levels of carbon monoxide (CO) emissions is unknown. The objective of this ecological study was to test the relationship by investigating whether areas of Australia with fewer noxious vehicles per capita experienced lower rates of MVEG suicide.

**Methods and Findings:**

We merged data on MVEG suicides in Australia (2001–06) with data on the number and age of vehicles in the national fleet, as well as socio-demographic data from the national census. Poisson regression was used to analyse the relationship between the incidence of suicide within two levels of geographical area—postcodes and statistical subdivisions (SSDs)—and the population density of pre-1986 and pre-1999 passenger vehicles in those areas. (There was a mean population of 8,302 persons per postcode in the study dataset and 87,413 persons per SSD.) The annual incidence of MVEG suicides nationwide decreased by 57% (from 2.6 per 100,000 in 2001 to 1.1 in 2006) during the study period; the population density of pre-1986 and pre-1999 vehicles decreased by 55% (from 14.2 per 100 persons in 2001 to 6.4 in 2006) and 26% (from 44.5 per 100 persons in 2001 to 32.9 in 2006), respectively. Area-level regression analysis showed that the suicide rates were significantly and positively correlated with the presence of older vehicles. A percentage point decrease in the population density of pre-1986 vehicles was associated with a 6% decrease (rate ratio [RR] = 1.06; 95% confidence interval [CI] 1.05–1.08) in the incidence of MVEG suicide within postcode areas; a percentage point decrease in the population density of pre-1999 vehicles was associated with a 3% decrease (RR = 1.03; 95% CI 1.02–1.04) in the incidence of MVEG suicide.

**Conclusions:**

Areas of Australia with fewer vehicles predating stringent CO emission laws experience lower rates of MVEG suicide. Although those emission laws were introduced primarily for environmental reasons, countries that lack them may miss the benefits of a serendipitous suicide prevention strategy.

*Please see later in the article for the Editors' Summary*

## Abstract 2

In an ecological study, David Studdert and colleagues show that areas of Australia with fewer vehicles pre-dating stringent carbon monoxide emission laws have lower rates of suicide due to asphyxiation by motor vehicle exhaust gas.

## Editors' Summary

**Background:**

Suicide (self-inflicted death) is a major, preventable public-health problem. About 1 million people die each year from suicide and about 20 times as many people attempt suicide. Globally, suicide rates have increased by nearly a half over the past 45 years and suicide is now among the three leading causes of death in people aged 15–44 years. Within this age group, 1 in 20 deaths is a suicide. Most people who commit suicide have a mental illness, usually depression or substance abuse, but suicide can also be triggered by a stressful event such as losing a partner. Often warning signs are present—a person who talks about killing themselves must always be taken seriously. Adequate prevention and treatment of mental illness and interventions that teach young people coping skills and improve their self-esteem have shown promise in reducing suicide rates, as have strategies (for example, restrictions on the sale of pain killers) that reduce access to common methods of suicide.

**Why Was This Study Done?:**

In Australia, the suicide rate has been declining since 1997 when a record 2,722 suicides occurred. Fewer suicides by asphyxiation (oxygen deprivation) by motor vehicle gas exhaust (MVEG) account for much of this decline. MVEG contains carbon monoxide, a toxic gas that blocks oxygen transport around the body. Although MVEG suicide is still the second most common means of suicide in Australia, its incidence has dropped by two-thirds since 1997 but why? One possibility is that national laws passed in 1986 and 1999 that lowered the permissible level of carbon monoxide in vehicle exhaust for environmental reasons have driven the decline in MVEG suicides. Evidence from other countries suggests that this might be the case but no-one has directly investigated the relationship between MVEG suicide and the use of vehicles with reduced carbon monoxide emissions. In this ecological study (a study in which the effect of an intervention is studied on groups of people rather than on individuals), the researchers ask whether the number of pre-1986 and pre-1999 vehicles within particular geographic areas in Australia is correlated with the rates of MVEG suicide in those areas between 2001 and 2006.

**What Did the Researchers Do and Find?:**

The researchers obtained data on MVEG suicides from the Australian National Coroners Information System and data on the number and age of vehicles on the road from the Australian Bureau of Statistics. MVEG suicides dropped from 498 in 2001 to 231 in 2006, they report, and 28% of passenger vehicles registered in Australia were made before 1986 in 2001 but only 12% in 2006; the percentage of registered vehicles made before 1999 fell from 89% to 60% over the same period. The researchers then used a statistical technique called Poisson regression to analyze the relationship within postcode areas between the incidence of MVEG suicide and the presence of pre-1986 and pre-1999 vehicles. This analysis showed that in areas where older vehicles were more numerous there were more MVEG suicides (a positive correlation). Specifically, the researchers calculate that if the proportion of pre-1986 vehicles on the road in Australia had stayed at 2001 levels throughout their study period, 621 extra MVEG suicides would have occurred in the country over that time.

**What Do These Findings Mean?:**

These findings show that in areas of Australia that had fewer vehicles on the road predating stringent vehicle emission laws, there were lower rates of MVEG suicide between 2001 and 2006. Unfortunately, this study cannot provide any information on the actual age of vehicles used in MVEG suicides or on the relationship between vehicle age and attempted MVEG suicides. It also cannot reveal whether those areas that had the sharpest decreases in the density of older vehicles had the sharpest decreases in suicide rates because very few suicides occurred in most postcodes during the study. Most importantly, the design of this study means that the researchers cannot discount the possibility that the changes in Australia's emission laws have steered people towards other methods of taking their own lives. Nevertheless, the findings of this study suggest that the introduction of stringent vehicle emission laws for environmental reasons might, serendipitously, be a worthwhile long-term suicide prevention strategy in countries where MVEG suicide is common.

**Additional Information:**

Please access these Web sites via the online version of this summary at http://dx.doi.org/10.1371/journal.pmed.1000210.

- Another *PLoS Medicine* research article, by Shu-Sen Chang and colleagues, investigates the evolution of the epidemic of charcoal-burning suicide in Taiwan
- The US National Institute of Mental Health provides information on suicide and suicide prevention
- The UK National Health Service Choices Web site has detailed information about suicide and its prevention
- The World Health Organization provides information on the global burden of suicide and on suicide prevention (in several languages)
- MedlinePlus provides links to further resources about suicide (in English and Spanish)
- Suicide Prevention Australia is a nonprofit, nongovernmental organization working as a public-health advocate in suicide prevention
- The Australian Institute of Health and Welfare has recently published a review of suicide statistics in Australia
- The National Coroners Information System is a database contains information on every death reported to an Australian coroner since July 2000 (January 2001 for Queensland)

## Introduction

In 2004, suicide accounted for an estimated 1.4% of deaths and 1.9% of years of life lost worldwide; among persons aged between 15 and 44 y, those figures were 5.2% and 5.5%, respectively [1]. While suicide rates continue to rise globally, Australia, which has long had one of the highest rates of suicide among developed countries, has experienced a remarkable decline in recent years.

After peaking in 1997, the Australian national suicide rate fell by 41% in the decade to 2006 [2]. Declines occurred among all major methods except hanging, but the steepest decline by far was in the incidence of asphyxiation by motor vehicle exhaust gas (MVEG), which fell by 67% between 1997 and 2006 [2]. Because MVEG is the second most common method of suicide, its decline has had a major impact at the population level: 40% of the decrease in Australia's overall suicide rate stems from the plunge in MVEG suicides.

Why MVEG suicides have fallen so dramatically is unclear. The most plausible explanation relates to changes in vehicle design that have reduced levels of carbon monoxide (CO) emissions, the fatal component of exhaust gas. Beginning in 1986, all new petrol-powered passenger vehicles sold in Australia were required to have catalytic converters in their engines; the same design law mandated a reduction in CO emission levels from 24.2 g/km to 9.3 g/km [3]. A subsequent design law, phased in between 1997 and 1999, further lowered the CO limit to 2.1 g/km [4].

Several previous studies in Australia [5],[6], the United States [7], and the United Kingdom [8]–[10] have explored the relationship between lower vehicle emissions standards and MVEG suicides. Their findings are mixed. But with one exception these studies consist of uncontrolled comparisons of suicide rates over time. The only controlled analysis [7] to date examined trends in MVEG suicide in the US over 30 y but did not focus specifically on the impact of reduced CO emissions. We conducted an ecological analysis of the relationship between pre-1986 and pre-1999 vehicles and rates of MVEG suicide in Australia.

## Methods

### Ethics Statement

The study was reviewed by the Human Research Ethics Committee of the Victorian Department of Justice (Melbourne, Australia).

### Data Sources

Data for this study came from three sources: the National Coronial Information System (NCIS), and the motor vehicle census and the national census, which are both conducted by the Australian Bureau of Statistics (ABS).

#### NCIS

NCIS is a national system of information and supporting infrastructure designed for use by coroners, researchers, and others interested in the prevention of injury and disease [11]. The dataset captures details of deaths reported to coroners in Australia, including the causes and circumstances of those deaths and characteristics of the deceased. Reporting of suicides and suspected suicides to the coroner is mandatory in every state and territory.

Data entry into NCIS is guided by detailed coding protocols [12] and quality assurance procedures [11],[13]. The reliability of the dataset has been tested in several previous studies and demonstrated very good consistency with official national statistics [14],[15]. With respect to suicides, NCIS figures are probably more accurate than official statistics compiled by the ABS, which must be finalised in some cases before the coroner's investigation of cause of death is complete [16],[17]. NCIS also contains greater detail on the nature and cause of deaths than any other national mortality datasets, making it an excellent source of information for tracking the frequency of specific types of fatal injury.

#### Motor vehicle census

To produce official estimates of the size and composition of the national vehicle fleet, the ABS has conducted a motor vehicle census periodically since 1971, and annually since 1995 (except in 2000) [18]. This census compiles data on vehicles registered with motor vehicle registration authorities in all states and territories. Vehicles under registration on the survey date, or whose registrations expired less than 1 mo before the survey date, are counted. The survey also obtains information on each vehicle included in the count, including its type (passenger, light commercial, truck, etc.), year of manufacture, and owner's residential postcode.

#### Census of population and housing

Australia's national census is administered by the ABS [19]. It has been conducted every 5 y since 1961. The census counts all persons who spend census night in Australia, with the exception of foreign diplomats and their families. Participation is mandatory. In addition to basic enumeration, the census collects a wide range of socio-demographic data on individuals and families.

### Study Dataset

We merged data from the three sources by postcode and year to produce a dataset at the postcode-year level. A small number of suicides (1.8%, 40/2,255) had to be dropped during the merge because the deceased's postcode was missing or did not match census postcodes. The NCIS provided counts of MVEG suicides in each postcode-year. The motor vehicle census permitted calculation of the total number of passenger vehicles manufactured prior to 1999 and prior to 1986 in each postcode-year. (The postcode in the NCIS refers to the deceased's residence; the postcode in the motor vehicle census refers to the registered owner's residence.) We converted these counts to incidence (suicides) and density (vehicles) measures by dividing them by the total population in the relevant postcode-year. The vehicle density statistic indexes the number of pre-1999 and pre-1986 vehicles to population counts at the area level. This is a more appropriate measure of the exposure of populations to noxious vehicle emissions than alternative measures, such as the proportion of older vehicles in an area's fleet.

We also used data from the national census to calculate the distribution of selected socio-demographic characteristics in each postcode-year. Specifically, we created variables describing percentages of the population that fell into designated categories of age, educational attainment, employment status, marital status, religion, country of birth, race, and income. We then controlled for these factors in examining the relationship between suicide and vehicle density because they are recognised or hypothesised predictors of suicide [20],[21], and thus have the potential to confound the relationship of interest.

Although the suicide and motor vehicle data were available for all years in the study period, the national census was conducted only in the first and last years. We therefore applied values from the 2001 census to study years 2001–03 and values from the 2006 census data to study years 2004–06. The final analytical dataset consisted of 13,752 postcode-years with an average of 2,292 postcodes per year. The mean population per postcode was 8,302 persons (interquartile range = 1,016–12,326 persons).

### Descriptive and Multivariate Analyses

We generated descriptive statistics on MVEG suicides in the sample, using the detailed information available in NCIS. We also plotted trends in the incidence of MVEG suicide and the population density of pre-1986 and pre-1999 passenger vehicles.

We used Poisson regression to analyse the relationship between MVEG suicide and the presence of the older vehicles. The dependent variable was the MVEG suicide count and the independent variables of interest were the population density of pre-1986 vehicles and pre-1999 vehicles (examined in separate models.) The unit of analysis was postcode-year and the models corrected standard errors for clustering within postcodes. The population for each postcode was declared as an offset term in the regression equation, allowing the suicide rate to be modelled as a linear function (on the logarithmic scale) of the vehicle density variables. The models also controlled for the various socio-demographic factors listed above and for time by including year as a categorical variable.

### Sensitivity Analyses

To test the robustness of our multivariate analysis to alternative specifications, we conducted three types of sensitivity analysis.

First, we reconstituted the dataset at the level of statistical subdivision (SSD)-year, re-ran the regression models, and compared results of the SSD-year version with the postcode-year version. SSDs, one of five main hierarchical levels in the Australian Geographic Classification System, are a general purpose spatial unit of intermediate size [22]. Their boundaries are based on socially and economically homogeneous regions characterised by identifiable links between the inhabitants. SSDs cover the territory of Australia without gaps or overlaps and take in much larger areas and populations than postcodes. There were 207 SSDs in Australia in 2001 and 217 SSDs in 2006. Using concordance files made available by the ABS, we transformed the dataset from the postcode-year level to the SSD level. Postcodes that spanned more than one SSD (14%, 350/2,515) were assigned to the SSD that covered more than 70% of its area. Postcodes with 70% or less of their area covered by a single SSD (4%, 89/2,515) were dropped. The final SSD-level dataset consisted of 1,236 SSD-years with a mean of 87,413 persons per SSD (interquartile range = 23,146–114,369 persons).

Second, 2.1% (284/13,752) of postcode-years had two suicides and 0.5% (62/13,752) had three or more suicides. We re-ran the main multivariable models excluding postcode-years with multiple suicides to test the impact of these outlier values on our findings.

Third, the approach used to adjust for postcode-level clustering in our original regression models was based on robust variance estimation. We tested an alternative to examine the effect of residual heterogeneity in suicide rates between postcodes. Specifically, we re-ran both the pre-1986 and pre-1999 Poisson regressions using a generalised linear mixed model with a random intercept at the postcode level.

## Results

### MVEG Suicides

83% of persons who suicided by MVEG between 2001 and 2006 were male (Table 1). Approximately one-fifth were divorced or separated and one-third were retirees, pensioners, or unemployed. The annual frequency of MVEG suicides fell by 54% in the study period, from 498 in 2001 to 231 in 2006. Victoria, New South Wales, and South Australia accounted for 70% of MVEG suicides.

**Table 1:** Characteristics of suicides by MVEG in Australia, 2001–06 (*n* = 2,255).

Characteristics	*n*	*Percent*
**Male**	1,872	83
**Age (y)**		
<18	12	1
18–45	1,298	58
46–64	729	32
65+	215	10
Unknown	1	<1
**Marital status**		
Married	753	33
Divorced or separated	484	21
Never married	482	21
Widowed	65	3
Unknown	471	21
**Employment status**		
Employed	1,214	54
Retired/pensioner	365	16
Unemployed	347	15
Other	103	5
Unknown	226	10
**Notification year**		
2001	498	22
2002	427	19
2003	393	17
2004	382	17
2005	324	14
2006	231	10
**State jurisdiction**		
Victoria	601	27
New South Wales	488	22
South Australia	487	22
Western Australia	285	13
Queensland	235	10
Tasmania	104	5
Northern Territory	42	2
Australian Capital Territory	13	1

### Trends in MVEG Suicides and Older Vehicles

Vehicles manufactured before 1986 constituted 28% of all passenger vehicles registered in Australia in 2001; by 2006, this had fallen to 12%. The percentage of passenger vehicles manufactured before 1999 declined from 89% to 60% over the period.

**Figure 1:**
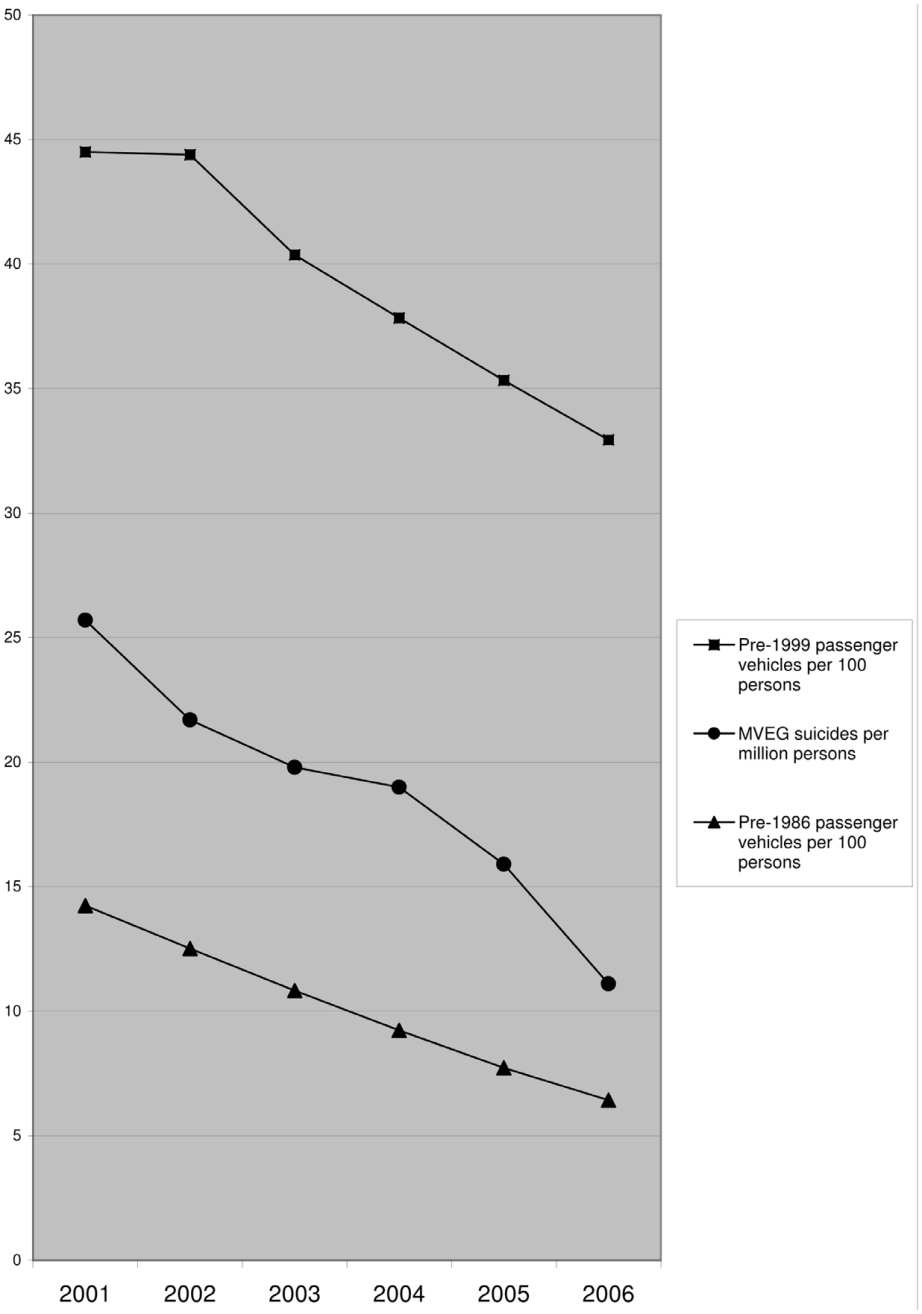
Trends in suicides by MVEG and population density of passenger vehicles manufactured before 1999 and 1986, respectively.

In Figure 1, the middle line shows a 57% decrease in MVEG suicides per million persons during the study period (from 25.7 per million in 2001 to 11.1 per million in 2006). The lower and upper lines in Figure 1 show changes in the population density per 100 persons of passenger vehicles manufactured before 1986 and 1999; respectively, they decreased by 55% (from 14.2 per 100 persons in 2001 to 6.4 in 2006) and 26% (from 44.5 per 100 persons in 2001 to 32.9 in 2006).

### Analytical Sample

Table 2 shows the means and standard deviations for all variables used in the post-code year analysis. There was an average of 16.1 suicides per 100 postcode-years. The population density of pre-1986 and pre-1999 vehicles averaged 10.2% and 39.2%, respectively.

**Table 2:** Characteristics of the postcode-years in analytical sample (*n* = 13,752).

Characteristics	Mean	SD
**MVEG suicides (per 100 postcode-years)**	16.1	45.8
**Pre-1986 passenger vehicles (per 100 vehicles)**	19.4	8.9
**Pre-1999 passenger vehicles (per 100 vehicles)**	74.9	13.1
**Pre-1986 passenger vehicles (per 100 persons)**	10.2	5.5
**Pre-1999 passenger vehicles (per 100 persons)**	39.2	12.3
**Age (%)**		
<25 y	32.8	5.5
25–64 y	53.5	4.7
65+ y	13.3	5.5
**Final year of school (%)**		
Year 10 or below	47.6	14.1
Year 11 or 12	49.9	14.5
**Employment status (%)**		
Employed	74.8	10.9
Unemployed	4.7	2.4
**Marital status (%)**		
Married	57.7	9.5
Divorced or separated	11.9	3.2
Widowed	6.4	2.7
Never married	33.0	9.6
**Christian (%)**	66.2	10.5
**Born in Oceania (%)** a	79.7	11.3
**Aboriginal or Torres Strait Islander (%)**	3.4	8.5
**Income (%)** b		
Low	29.9	7.6
Middle	42.0	7.5
High	26.3	10.7

a Includes Australia and New Zealand.

b Low income is defined as average gross weekly income in the postcode–year less than AUD$200 per week in 2001–03 and less than AUD$250 per week in 2004–06; middle income is AUD$200–AUD$599 per week in 2001–03 and AUD$250–AUD$799 per week in 2004–06; high income is AUD$600 or more per week in 2001–03 and AUD$800 or more per week in 2004–06. The mean values shown refer to the percentage of all residents over 19 y whose income level falls into this classification.

### Multivariate Analysis

The regression analysis showed a significant positive relationship between rates of MVEG suicide and the population density of both pre-1986 vehicles (rate ratio [RR] = 1.06; 95% confidence interval [CI] 1.05–1.08; *p*<0.001) and pre-1999 vehicles (RR = 1.03; 95% CI 1.02–1.04; *p*<0.001) (Table 3). Put in the context of declining rates, a percentage point decrease in the population density of pre-1986 vehicles was associated with a 6% (95% CI 5%–8%) decrease in the MVEG suicide rate and a percentage point decrease in the population density of pre-1999 vehicles was associated with a 3% decrease (95% CI 2%–4%) in the MVEG suicide rate.

**Table 3:** Multivariate regression results: Relationship between population density of passenger vehicles manufactured before 1986 and 1999, respectively, and rates of suicide by motor vehicle exhaust.

Predictors^a^	Pre-1986 vehicles			Pre-1999 vehicles		
	RR	95% CI	*p*-Value	RR	95% CI	*p*-Value
**Population density of older vehicles**	1.06	1.05–1.08	<0.001	1.03	1.02–1.04	<0.001
**Age (y)**						
0–24	1.00	0.98–1.03	0.66	1.00	0.98–1.02	0.88
65+	1.00	0.97–1.03	0.84	0.99	0.95–1.02	0.37
**Year 10 or below**	1.01	1.00–1.02	0.16	1.01	1.00–1.01	0.26
**Unemployed**	1.02	0.98–1.06	0.36	1.02	0.98–1.07	0.28
**Marital status**						
Divorced or separated	1.03	1.00–1.06	0.04	1.02	1.00–1.05	0.07
Widowed	0.98	0.94–1.04	0.54	0.99	0.94–1.04	0.70
Never married	0.99	0.98–1.00	0.03	0.99	0.98–1.00	0.06
**Christian**	0.98	0.97–0.99	<0.001	0.98	0.97–0.98	<0.001
**Born in Oceania**	1.01	1.00–1.01	0.04	1.01	1.01–1.02	<0.001
**Aboriginal or Torres Strait Islander**	1.00	0.98–1.01	0.92	1.00	0.99–1.02	0.59
**Income**						
Low	0.97	0.95–0.99	0.002	0.98	0.96–0.99	0.01
High	0.99	0.98–1.00	0.15	0.98	0.97–1.00	0.007

Not shown are continuous variables included in the regression models that indicated the percentages of the population in each postcode-year for whom, respectively, educational status, employment status, region of birth, Aboriginal or Torres Strait Islander race, and income level were not known. None of those variables was statistically significant. Both models also controlled for year using dummy variables.

a The reference categories are: 25–64 y (age); year 11 or 12 (final year of education); employed; married; non-Christian; born outside Oceania; not Aboriginal or Torres Strait Islander; and middle income.

Several other predictors were significant. The percentages of Christians, low income earners, and adults who had never married were negatively correlated with MVEG suicide. The percentage of residents born in the Oceania region (which includes Australia), on the other hand, was positively correlated with MVEG suicide. Age, unemployment, low education, and Aboriginal and Torres Strait Islander race were not significant predictors of the MVEG suicide rate in either model. The percentage of adults who were divorced or separated was positively correlated with the suicide rate in both models, although its statistical significance was borderline.

### Sensitivity Analyses

Reconstitution and reanalysis of the dataset at the SSD-year level produced similar results to the postcode-year analysis. The vehicle density variables remained significant predictors the MVEG suicide rate in both the pre-1986 model (RR = 1.07; 95% CI 1.04–1.09, *p*<0.001) and the pre-1999 model (RR = 1.04; 95% CI 1.03–1.06, *p*<0.001) in the SSD analyses, with slightly larger coefficients than in the postcode-year analyses.

Reruns of the pre-1999 model after exclusion of postcode-years with two or more suicides (*n* = 284) and three or more suicides (*n* = 62) did not change the size of the coefficients on the vehicle density variables (to the second decimal place) or their statistical significance. Reruns of the pre-1986 model with these same exclusions increased fractionally the coefficient on the vehicle density variable (for both types of exclusions, RR = 1.07, 95% CI = 1.05–1.08).

The estimates in our main models were quite robust to reestimation in a generalised linear mixed model with a random intercept at the postcode level. The coefficients on the vehicle density variable were slightly smaller in the pre-1986 (RR = 1.05) and pre-1999 (RR = 1.03) models, but retained significance at the *p*<0.001 level.

## Discussion

This study found a strong positive association between the area-level prevalence of older cars and rates of suicide by MVEG in Australia. In 1986 and 1997–99, new environmental laws took effect in Australia requiring all passenger vehicles sold to emit substantially lower levels of CO than had previously been permissible. Because the mandates applied to new cars, their impact took time to penetrate the national fleet, and the speed with which the newer, safer cars replaced older, noxious ones varied across communities.

Our estimates suggest that if the proportion of pre-1986 (pre-1999) vehicles in the national fleet had remained at 2001 levels throughout the study period, 621 (366) more MVEG suicides would have occurred in Australia. These estimate come from a postcode-year level multivariable model that predicts the number of suicides over the period 2001–06, using each of the actual values for covariates in those years, except the vehicle density measure, which is fixed at its 2001 value across all postcode-years. The difference between the total number of suicides predicted in the pre-1986 (*n* = 2,836) and pre-1999 (*n* = 2,581) version models and the actual number (*n* = 2,215) estimates the “excess” suicides, or lives “saved,” associated with the introduction of vehicles with lower CO emissions. Extending beyond the 2001–06 period would, of course, increase the extrapolations, but there are important caveats. First, the figures should not be interpreted as additive because pre-1986 vehicles are a subset of pre-1999 vehicles. Second, given that our models explained only part of the area-level variability in MVEG suicide rates, these estimates of population impact should be interpreted with caution.

The US led the transition to lower CO emissions, especially the state of California [23]. With the introduction of catalytic converters to vehicles in 1975, limits were set at 9.2 g/km, and then lowered to 2.1 g/km in 1981. Mott and colleagues [7] found a 43% decline in the MVEG suicide rate between 1968 and 1998. Most of the reduction occurred in the 1990s, at least 15 y after the first major changes in emission laws, but the analysis did not attempt to link this reduction directly to those changes. Hence, the impact on suicide rates from the country's pioneering efforts in lowering CO emissions levels has not been assessed directly.

European countries intervened relatively late [23]. The European Union did not adopt regulations requiring catalytic converters until 1993. In the UK, for example, CO limits for passenger vehicles decreased to 2.72 g/km in 1993. More recent “Euro” design standards have virtually eliminated CO from emissions. Several studies have suggested that these changes have contributed to a reduction in MVEG suicide in the UK [8]–[10], particularly among young men [24], and in Denmark [25],[26]. However, these studies use uncontrolled before-and-after statistics to reach that conclusion and do not quantify the effect.

In Australia, evidence from previous research is also limited. An analysis of 100 MVEG suicides in Victoria between 1994 and 1996 found no relationship between the 1986 emission law and MVEG suicides [5]. Mott and colleagues' results [7] suggest that it may have been too early to find a relationship if it existed, especially considering the average vehicle life in Australia is nearly 3 y longer than in the US [27]. A second study [6] analysed the manufacture year of vehicles used in 90 MVEG suicides in Victoria in 2002; it found disproportionately few of the vehicles were built between 1986 and 1997, relative to the proportion of cars of that age in the state's fleet, and even fewer were built before 1986. Again, however, the study was not designed to measure the population impact of the emission laws; the data also came from a single year and state.

Our study is, to our knowledge, the first controlled analysis of the relationship between vehicle age and incidence of MVEG suicide, and also the first to compare differences in suicide rates across populations with varying “exposures” to noxious vehicles. Our findings suggest that the reductions in CO emissions levels in Australia are an important explanation for the sharp decline in national rates of MVEG suicide.

More generally, this study joins a body of previous research suggesting that means restriction may help to prevent suicide [20],[28],[29]. For example, the detoxification of domestic coal gas in the Great Britain [30],[31] and Japan [32] was linked to declines in suicide by gas poisonings in those countries. Similarly, laws mandating blister packs [33] for analgesics and restricting the amount of analgesia available in a single purchase [34],[35] have both been associated with lower rates of overdoses.

The perennial question for means restriction, however, is whether it actually averts fatalities, or merely steers people to alternative methods. Amos and colleagues' examination [9] of the decline in MVEG suicides in England and Wales, for example, concluded that it was partially offset in young people by method substitution, particularly hanging. Similarly, a reexamination of the British coal gas story suggests substantial method substitution among younger people [31]. Although our study design did not allow investigation of substitution effects, simple trend data are not consistent with a counterbalancing of the decline in use of MVEG by reversion to other methods. There are no commensurate spikes in other methods [2]. Indeed, except for hanging, which has changed little, the incidence of suicide by all methods has been declining since the late 1990s, though not as sharply as MVEG.

Our study has several limitations. First, ecological analyses have a variety of intrinsic constraints [36]—most notably, without unit record data there is no direct way to identify and adjust for individual-level confounding. Second, some vehicles, particularly those imported from countries with stricter standards, may have conformed to lower emissions levels than the limit in force in Australia. We could not gauge such preemptive activity, but to the extent it occurred it seems unlikely to have done so according to some systematic pattern across areas. Third, our data sources did not capture information about the age of the vehicles used in the suicides. Although this additional information may shed further light on the relationship between the design changes and suicide rates, it would not be dispositive unless information on attempts was also examined. Fourth, our study focused on suicide completions, not attempts. It may be helpful to analyse attempt data in future research in this area, although the weaknesses of attempt data have been extensively analysed and discussed [37].

A fifth limitation is that we could not extend the analysis to determine whether areas with the sharpest decreases in the density of older vehicles also experienced the sharpest decreases in suicide rates. The analytical barrier stems from the rarity of MVEG suicide at the population level. A majority (87%) of postcodes-years and a substantial minority (40%) of SSD-years had no MVEG suicides, and when one occurred, the rate deviation was often dramatic. Unfortunately, this data structure did not allow for meaningful comparisons of changes within areas (postcodes or SSDs) between early and later years of the study period. Aggregating at area levels larger than SSDs—for example, states—addresses that problem, but trades it for others—namely, the sample size becomes small and the ability to discriminate between areas on the vehicle measures is lost.

Although vehicle emissions reduction in Australia appears to have been a successful intervention in reducing suicide, it is an unusual suicide prevention strategy for a couple of reasons. First, the result is serendipitous. Emission laws were motivated primarily by environmental concerns; their impact in helping to reduce suicide has been a valuable but largely unintended social benefit. Second, by targeting new vehicles, emission laws represent a long-term suicide prevention strategy. The Australian and US experiences suggest that the impact on MVEG suicide rates may remain latent for years, until the penetration of newer vehicles into the national fleet reaches a certain point, and even then unfolds quite gradually. Other countermeasures, such as cabin sensors and modification of exhaust pipes to be incompatible with hose attachments [27], could save lives in the interim.

Findings from this study have wider implications. Many developing and middle-income countries have not yet enacted stringent vehicle emission laws [38],[39]. Other countries, including India, Russia, and China, have moved toward adoption of Euro standards, but relatively recently. In addition, the rigour with which vehicle emission standards are enforced is highly variable [40],[41]. For places in which low CO limits are not yet adopted, have been adopted late, or are not enforced, suicide prevention benefits may be missed.

## Abbreviations

CI: confidence interval
CO: carbon monoxide
MVEG: motor vehicle exhaust gas
RR: rate ratio
SSD: statistical subdivision
